# A New Method to Design and Manufacture a Low-Cost Custom-Made Template for Mandible Cut and Repositioning Using Standard Plates in BSSO Surgery

**DOI:** 10.3390/bioengineering11070668

**Published:** 2024-06-29

**Authors:** Liliana Di Brigida, Antonio Cortese, Emilio Cataldo, Alessandro Naddeo

**Affiliations:** 1 Department of Industrial Engineering, University of Salerno, Via Giovanni Paolo II, 132, 84084 Fisciano, SA, Italy; lilianadibrigida@libero.it (L.D.B.); e.cataldo@technodesign.it (E.C.); 2 Technogym Research and Development Department, Via Calcinaro 2861, 47521 Cesena, FC, Italy; 3 Department of Medicine, Surgery and Dentistry, University of Salerno, Via Salvador Allende, 84081 Baronissi, SA, Italy; ancortese@unisa.it; 4 Techno Design S.r.l., via Rosa Jemma, 2, 84091 Battipaglia, SA, Italy

**Keywords:** surgical template, low-cost procedure, standard plate, computer-aided surgery, orthognathic

## Abstract

In this study, a new methodology for designing and creating a custom-made template for maxillofacial surgery has been developed. The custom-made template can be used both for cutting and repositioning of the mandible arches for executing a BSSO (bilateral sagittal split osteotomy) treatment. The idea was developed in order to give the possibility of using a custom-made template with standard plates, thus reducing long times, high costs and low availability of custom-made plates; this represents the proof of novelty of the proposed template, based on a well-established methodology. The methodology was completely developed in the CAD virtual environment and, after the surgeons’ assessment, an in-vitro experiment by a maxillofacial surgeon was performed in order to check the usability and the versatility of the system, thanks to the use of additive manufacturing technologies. When computer-aided technologies are used for orthognathic surgery, there are significant time and cost savings that can be realised, as well as improved performance. The cost of the whole operation is lower than the standard one, thanks to the use of standard plates. To carry out the procedures, the proposed methodology allows for inexpensive physical mock-ups that enable the BSSO procedure to be performed.

## 1. Introduction

Over the decades, we have progressively and then increasingly witnessed a migration of traditional medicine and surgery towards computerised and computer-aided practices.

In any industrial or industrialised context, the necessary condition for the fruitful implementation of any project or product concerns the triad of resources, time and purpose; also, in this case, one has to consider the hospital system as an industrial system that must generate revenues while having to face costs. Therefore, we can give attention to the following consideration: the use of computer-aided technologies for orthognathic surgery entails notable advantages in terms of time and costs, with a view to reducing them when the system is fully operational, and improving performance. 

The reasons why people undergo orthognathic surgery cannot be debased and labelled as mere aesthetic reasons. The authors of [1] fully defined the motivations that push patients to undergo surgery: a combination of respiratory, masticatory and speech difficulties are the main reasons why patients can present for this surgery. It is therefore necessary to also consider the possible presence of episodes of bullying and a lack of self-esteem and anxiety. 

IT tools are crucial within the branch of medicine both from a clinical and surgical point of view; nowadays, in fact, we are seeing more and more robotic interventions, instrumental analysis with increasingly cutting-edge devices or interventions using increasingly computer-aided devices or approaches [2]. 

As [3] demonstrated, the virtual OGS (orthognathic surgery) planning method is feasible and more effective than the traditional approach at increasing the intuitiveness and for reducing errors and planning times.

Computer-aided practices contribute not only to the better repeatability of a process by having more precise data available, which, therefore, allows optimization of the model itself, but also to improvements in the definition of the implementation methodology; the data with different definitions of the parameters and different tools for their optimization can be appropriately processed and provide intermediate data for the process [4,5].

As mentioned above, in the field of orthognathic surgery, it is important to immediately specify the two substantial advantages regarding the computer-aided approach: improvements in performance and reductions in costs.

The path of orthognathic surgery towards being custom-made is a widely shared and appreciated approach, regardless of the nature and motivation of the operation. Custom-made benefits have been found in the computer-aided approach [6] as a valid response to the multiplicity of needs that vary depending on the specific cases; engineering solutions include custom-made plates, cutting guides, and osteogenesis distractors [7,8,9,10].

The authors of [11] considered the OPS (orthognathic positioning system) to be safe and well tolerated, providing positional control with considerable surgical accuracy. The OPS simplified surgery by being independent of support from the opposite maxilla and obviating the need for classic intermaxillary occlusal splints.

As expressed by [12], customized osteosynthesis plates for OS show good precision for the maxilla and greater variation in the mandible. These advances in the field of orthognathic surgery are intended to reduce time and effort for surgeons. 

The substantial limits that can be found in a traditional approach lie in the poor accuracy and predictability already present in the planning phase; therefore, computer-assisted virtual surgery has proved to be a valuable resource for producing predictable results.

CAD/CAM technologies have proven to be a very valuable tool to assist the surgeon in the operative and pre-operative phases, especially for what is the planning phase of the operation itself; in order to do this, it is essential that there is close collaboration between the medical team and an expert in these new computer-assisted technologies.

Virtual planning appears to be an accurate and reproducible method for planning orthognathic treatment [13].

The versatility of IT tools allows us to think of VSP and CAD/CAM technology as usable for different aspects, from the design and production of surgical tools to diagnostic reasons, rehearsing for complex surgical procedures and aid in pre-surgical planning [14].

Nowadays, there are numerous applications of additive technology in many fields; the medical branch is not excluded, and the techniques that have received the most attention are fused deposition modelling (FDM), inkjet printing, stereolithography and selective laser sintering (SLS) [15,16].

These days, filament wires are constructed from composite materials that have undergone a great deal of research, since their qualities are specifically designed to meet the mechanical, chemical and physical needs of biomedical applications. The composite material has no harmful effects or inflammatory properties, and it is made to resemble tissues or organs in in vivo settings [17].

While cutting production costs, three-dimensional (3D) printing technology expands the possibilities for patients to receive individualized care at a lower cost [18].

The main advantages of virtual surgical planning (VSP) or computer-aided surgical simulation (CASS) in orthognathic surgery facilitating the diagnosis, treatment planning and evaluation of the treatment outcomes of dentofacial deformities have been highlighted by [19]; VSP and CASS are essential for digital transfer methods such as computer-aided designed and manufactured (CAD/CAM) splints, customized mini-plates with cutting and drilling guides, and navigation.

The potential of studies and investigations on orthognathic surgery were highlighted by [20], who reported that VSP is the future for planning orthognathic surgery in terms of its high accuracy and time-efficiency; moreover, as the VSP program continues to evolve, research on how to reduce the time and cost of the work for each step should be carried out. 

In fact, progressive improvements in the hardware and software have made it easy to obtain 3D models [21]. Moreover, nowadays, additive manufacturing is a procedure that we can consider to be conventional. Therefore, it is much simpler to recreate three-dimensional models that perfectly reflect the morphology of the maxillary elements.

High-resolution low-dose computed tomography is useful for evaluating the facial skeleton and soft tissues after surgery, as well as for depicting a variety of possible complications [22], and these data can be also used to obtain 3D models for virtual verification after the surgeons’ activities.

Nowadays, VSP+CAD/CAM practices are extremely well established. Our goal was to indicate a design flow for defining a physical solution, i.e., a template, that obviously cannot be generic and unique but varies from patient to patient. The template presented in this article is a custom-made template, designed specifically for the patient; it is customized in that it helps to cut and to guide the repositioning of mandibular segments, but takes advantage of the use of standard implantable devices (plates and screws).

## 2. Materials and Methods

The creation of a custom-made surgical device implies several steps that are necessary for its modelling and creation. The proposed new methodology that allows the use of standard plates with a customized template is summarized in the following steps.

1. Acquisition of CT radiological images and evaluation of the diagnosis;
2. Segmentation of radiological images (DICOM to CAD format);
3. Mesh manipulation in a polysurface structure (IGES);
4. Making an osteotomy plan;
5. Planning the distraction;
6. CAD design of the custom-made template (for cutting and repositioning);
7. Identification of the location of the plate;
8. Identification of the plate holes on the osteotomized mandible;
9. Recomposition of the mandible with the holes and models overlapping;
10. Fine-tuning of the CAD model of the template;
11. Rapid prototyping of the template, mandible and plate, if needed;
12. Positioning the template on the mandible;
13. Creation of osteotomies and cutting of the screw heads;
14. Removal of the template, distraction with a plate and replacement of the screws.

**Figure 1:**
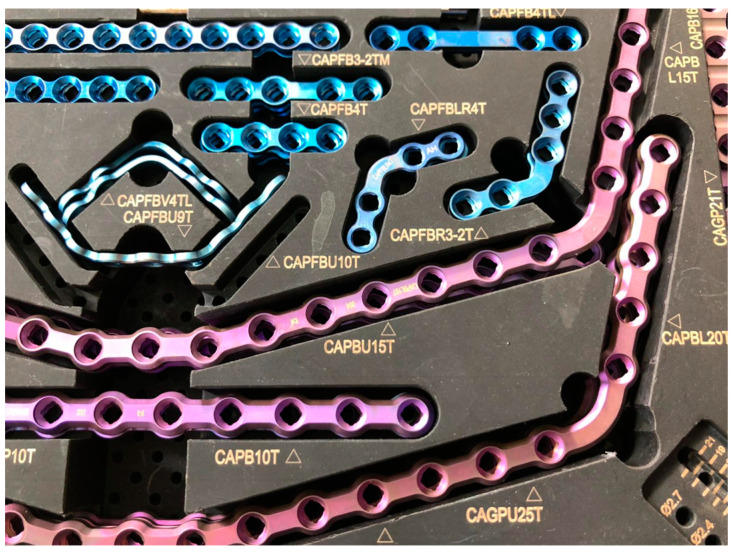
The standard Carcitek plate set by Tekka.

Each step of the methodology was developed in CAD environment and verified in an in vitro experiment. First of all, the suggested template’s architecture necessitates the replication of surgical situations, such as osteotomies, in a virtual setting with varying degrees of distraction; thus, the standard plate kit of the Carcitek set by Tekka (Global D) (Figure 1), used at the maxillofacial surgery ward of the hospital San Giovanni di Dio e Ruggi d’Aragona in Salerno, was recreated in the virtual environment.

In order to specify the osteotomies, the geometrical properties of the Stryker “Lindemann” drill were evaluated.

### 2.1. Reconstruction of the Mandibular Geometry

The initial step in template design is reconstruction of the maxillofacial region.

Firstly, computed tomography or magnetic resonance imaging can typically be used to scan the anatomical areas of interest. 

An anonymised real patient admitted to the San Giovanni di Dio e Ruggi d’Aragona hospital underwent a CT scan of the maxillofacial region. The scan produced 196 slices that were later used as input for a segmentation process through 3DSlicer, an open-source and multi-platform software package that is widely used for medical, biomedical and related imaging research [23].

As mentioned by [24], the primary processes of CAD/CAM and VSP technologies begin with data collection and proceed through to the design and production of individual implants, virtual surgical and treatment planning, and assessment of the outcome. 

Digital Imaging and Communications in Medicine (DICOM) data were imported as a folder, and the “volume rendering” module was simultaneously activated with a “pre-set” such that the bone elements were visible as the elements of interest.

The mandibular and jaw region was included in the ROI (region of interest), which was determined by the crop volume. The ROI needed to be seen as a cube with an upper face that was, in this example, positioned in three dimensions above the glenoid cavity and condyle.

The CT scanner used in this study was a Siemens Healthineers CT119680 model SOMATOM go.TOP, with Syngo CT VA30A Software, Kernel Br60f\3, with a tube voltage equal to 7.0 MHU. The characteristics of the acquired CT were as follows:

- Distance from the source to the detector (distance in mm from the source to the detector arrays) = 949.075;
- Distance from the source to the patient (distance in mm from the source to the isocentre or the centre of the FOV) = 541;
- Reconstruction diameter (diameter in mm of the region containing the data for image reconstruction) = 250;
- Thickness of the slice (mm) = 1.25;
- Spiral pitch factor (ratio between the advancement per rotation and the width of total collimation) = 0.5625 mm.

This fine scanning seems to be useless when developing a CAD model for 3D printing. Nevertheless, a high number of scanned points allowed us to develop a starting 3D-model that was very similar to the patient’s bone geometry and reduced the final cumulative error from reality to 3D printing, through scanning, segmentation, reconstruction and slicing. 

During the phase of processing an image, which can be executed with semi-automatic or automatic algorithms, attention must be paid to some aspects that would lead to a false representation of the elements of interest, particularly the following:

- Image noise and low contrast in the images, resulting in difficulty in identification of the tissue;
- Inhomogeneity of the tissue;
- Variability in the image caused by pathologies.

The patient’s CT scan was processed using the segment editor module of 3D Slicer. The threshold function was used to define four levels for:

- The jaw;
- The dental arches;
- The screws (foreign matter).

**Figure 2:**
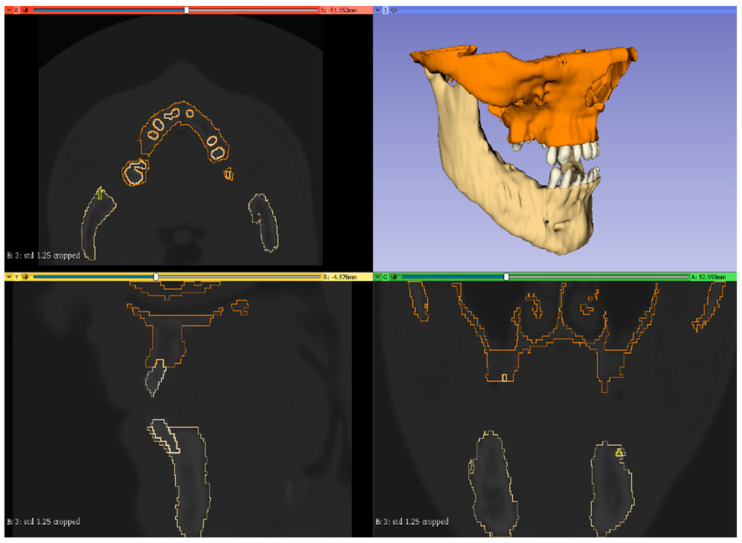
Segmentation operation.

The master volume, or the volume we wished to segment with one or more subsets, was defined within the “segment editor” module. Each subset could be itemized in terms of colour and name (Figure 2).

**Figure 3:**
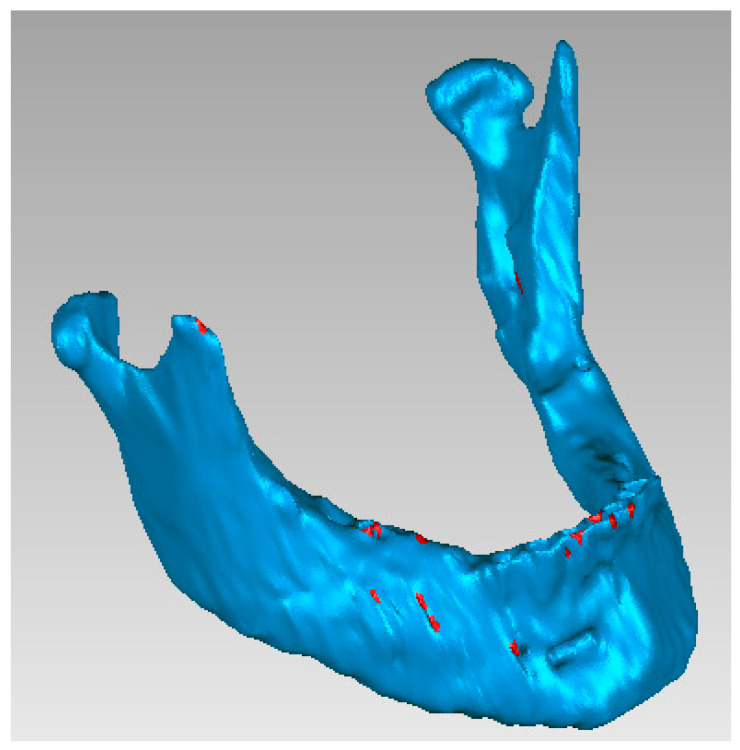
Configuration of the jaw before correction.

The .stl file from 3D Slicer had previously been imported into a commercial software package for mesh correction (Geomagic), through the use of the “feature recognition”, “mesh doctor” and “fill holes” tools, in order to be able to process the model of the mandible–maxilla–teeth assembly, both for the upper and lower arch, at a later stage. As one can see (in red), all the meshes that were defective or unsuitable were then corrected by the software (Figure 3).

This operation was carried out both for the .stl file of the mandible and for the teeth of the lower arch; once analysed and corrected, they were saved in IGES (initial graphics exchange specification) format.

Since the maxillary assembly has no effect on the template’s modelling stages, it was chosen to keep the 3D models that came from the 3D Slicer in .stl format to avoid increasing the processing time. 

Rhino 7.0 (Rhinoceros, s.d.), a commercial 3D modelling application, was utilized for the template’s design and modelling. 

Since the mandible’s .igs file contained 864 poly-surfaces, it could be treated as a single object in Rhino 7 after being imported using the Join command. 

**Figure 4:**
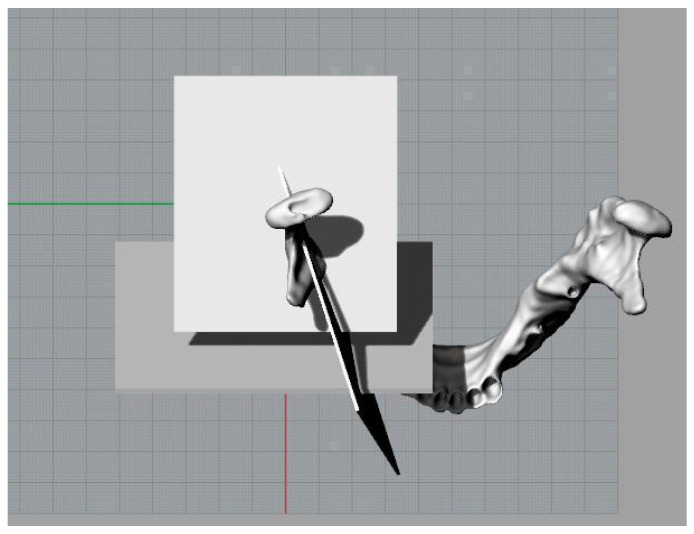
Three-dimensional model of the mandible and cutting planes to simulate osteotomies.

Three parallelepipeds were defined with a depth equivalent to the thickness of the drill used for this kind of intervention in order to replicate the osteotomies (Figure 4).

**Figure 5:**
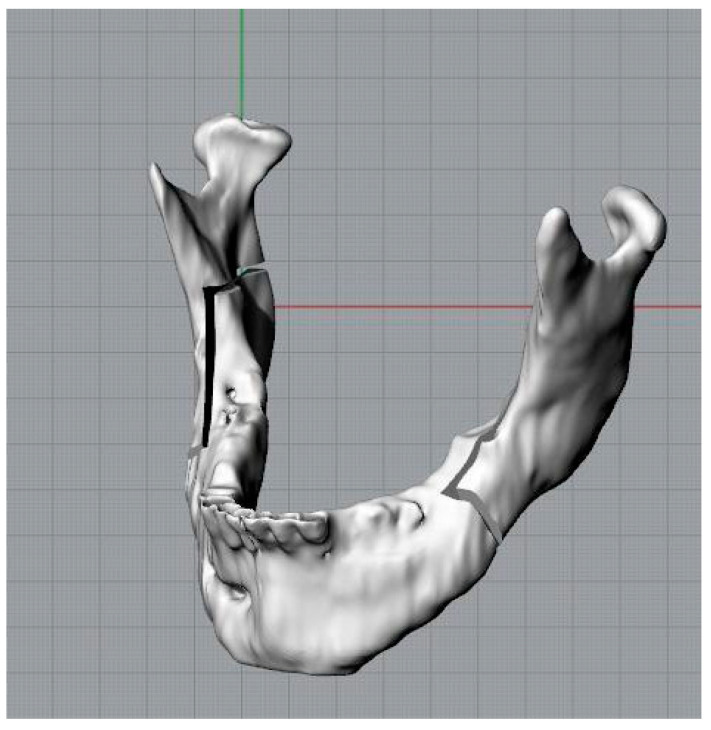
Osteotomized mandible.

A Boolean subdivision operation was performed, with the resulting removal of the “bony” portion from the osteotomies, which was located between the mandible and the cutting planes, in order to replicate and visualize the osteotomies (Figure 5). Analogous procedures were likewise executed for the left branch.

**Figure 6:**
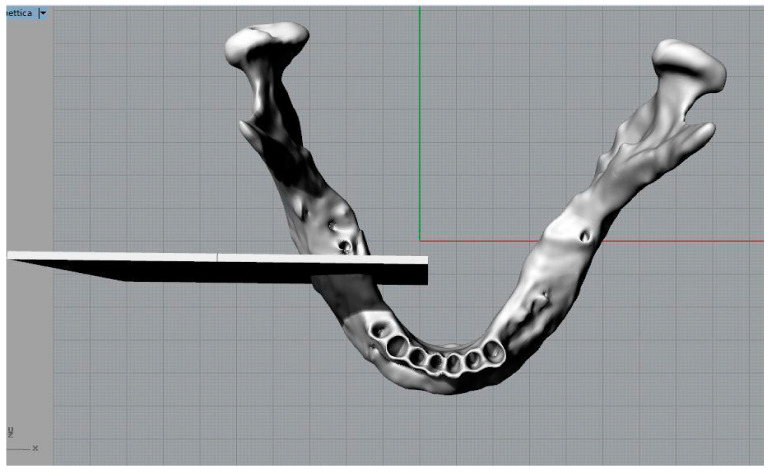
Mandible and cutting plane for the representation of the first osteotomy section.

The procedure that led to the visualization of the osteotomized mandible was as follows:

1. Creation of the “mandible” level and insertion of the mandible element;
2. Elimination of the levels by default;
3. Creation of the “cutting plane 1 right” level, selection of the level and design of the first cut through a parallelepiped with a width equal to the diameter of the drill used during the intervention (Figure 6).

A similar procedure was also carried out for Cutting Plane 2 and Cutting Plane 3.

The main steps were as follows:

1. Creation of the “cut mandible” level, representing the osteotomized mandible; on this level, the mandible previously duplicated on another virgin mandible level was copied;
2. Execution of the Boolean difference between the mandible and the three cutting planes;
3. Execution of the Boolean subdivision between the cutting planes (this operation was carried out to be able to combine all the segments subject to the osteotomy in a single level at a later stage);
4. Boolean union at the level of the mandible to obtain an external branch that included the condyle and the coronoid process, and a central branch;
5. Union of the cutting stump, as described in Step 6, and the creation of a new “right complete cut” level where the joined “s” abutment was copied.

In theory, in order to perform osteotomies on the left branch as well, one could proceed with a mirroring operation of the cutting planes, but it is not certain that there is perfect symmetry between the two mandibular rami; it is more appropriate to proceed following the procedure just described, taking the anatomical details of the case into consideration.

**Figure 7:**
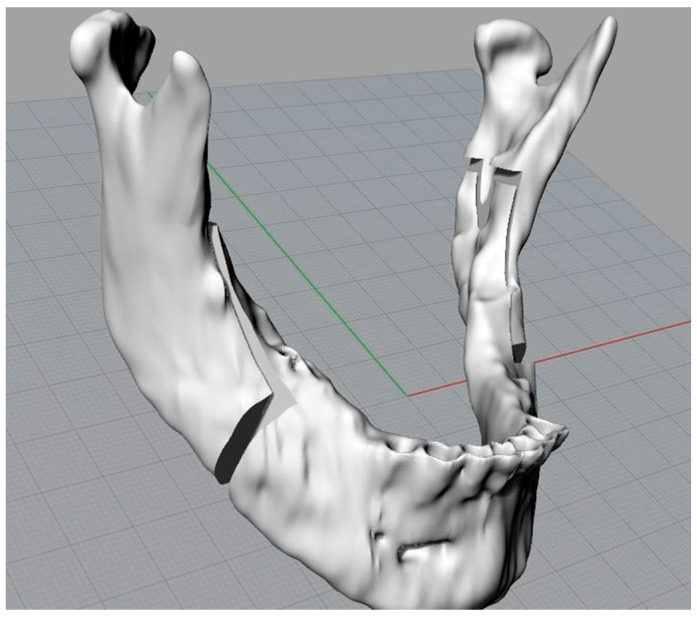
Jaw in the final, desired position.

The mandible, following these operations, will have three elements: two branches and a central body (Figure 7).

### 2.2. Modeling the Resection Template

In defining the workflow to design and create the template, the first 10 phases of the methodology, which can become a procedure, are carried out in a virtual environment with the aim of innovating the traditional procedure.

The 11th phase involves the actual creation of the template using additive technology; for the creation of the template, it is preferable to use SLS (selective laser sintering) for achieving a better resolution, but for our purposes, we used FDM (fused deposition modelling) technology.

The last three phases are those that must be implemented during surgery.

In the third phase, it is necessary to virtually identify the correct repositioning of the jaw and, indirectly, of the dental arches. In the case in question, a 3 mm advancement of the mandibular segment was set solely for the implementation of the workflow. The value of the advancement is just an example to develop the physical mock-up; due to the general approach to the problem, any kind of displacement of the mandibular segments, both in advancement and in retrogression, can be simulated and treated by the proposed template and methodology.

**Figure 8:**
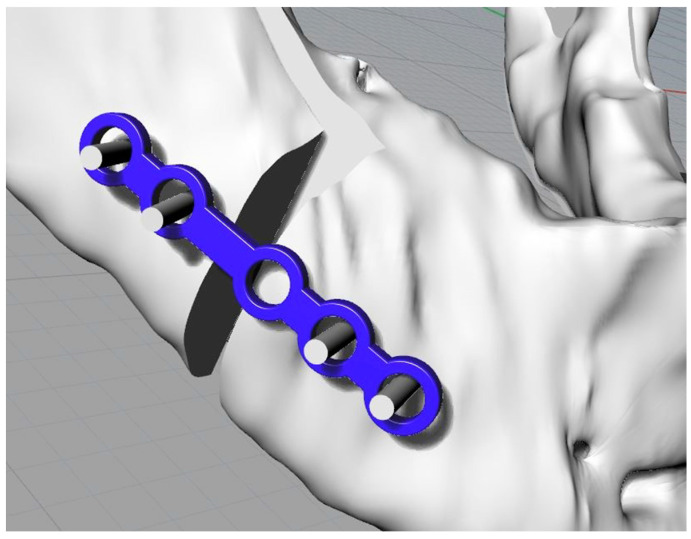
Jaw in the desired final position with the screws and the standard plate (in blue).

**Figure 9:**
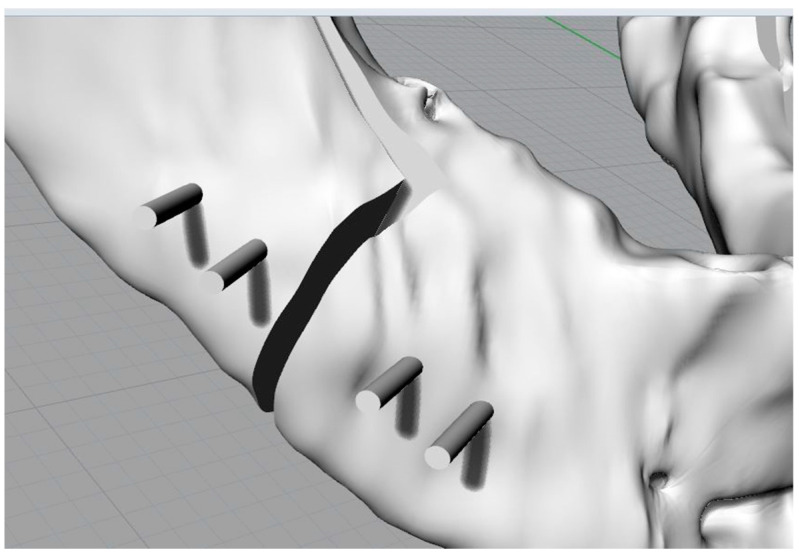
Osteotomised mandible with screws without heads (cylinders).

The mandible in the distracted configuration was used, together with the morphology and the distance between the centres of the plate, as previously presented, to identify the slots for the screws on the template. This is the key step in the template’s definition. The latter, in fact, being fixed to the jaw at specific points will allow the automatic repositioning of the jaw, thanks to the plate, in the desired configuration (Figure 8 and Figure 9).

At this point, the mandible is returned to its original position; however, one should always have the position of the screws on the proximal segment as a reference.

**Figure 10:**
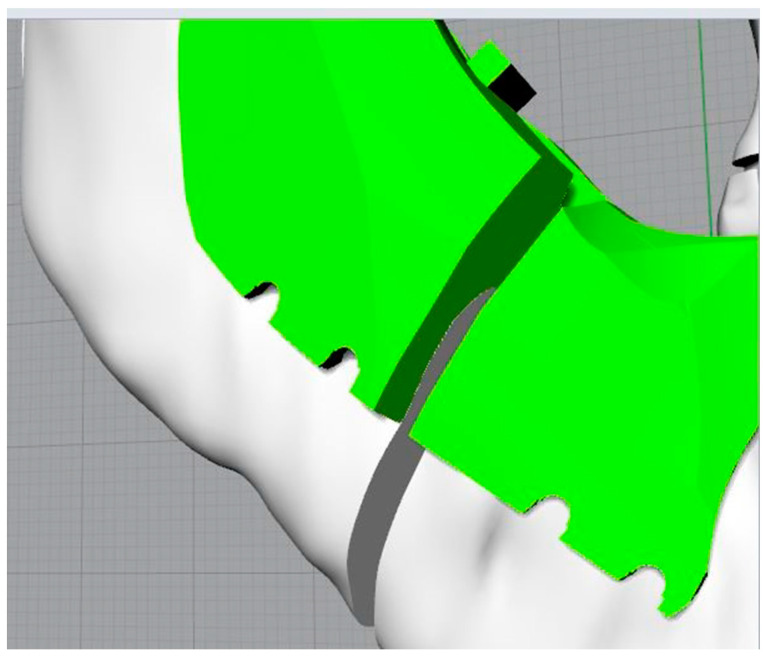
Details of the screws’ housing and grooves.

The design of the cutting and positioning template was carried out using the extrusion command of a closed curve; the solid element thus generated was first subjected to Boolean operations of subdivision and subtraction, both with the jaw and with the cutting planes identifying the osteotomies, then its shape was modelled to better adapt to the spaces available in the oral cavity (Figure 10). The coupling surface between the template and the mandible’s bones and teeth had to be perfectly modelled only in the real coupling parts (in this case, the anterior tooth and the rear arch of the mandible), while the other surfaces could be modelled by taking the presence of the mucoperiosteum layer into account, without changing the upper surfaces of the template itself.

**Figure 11:**
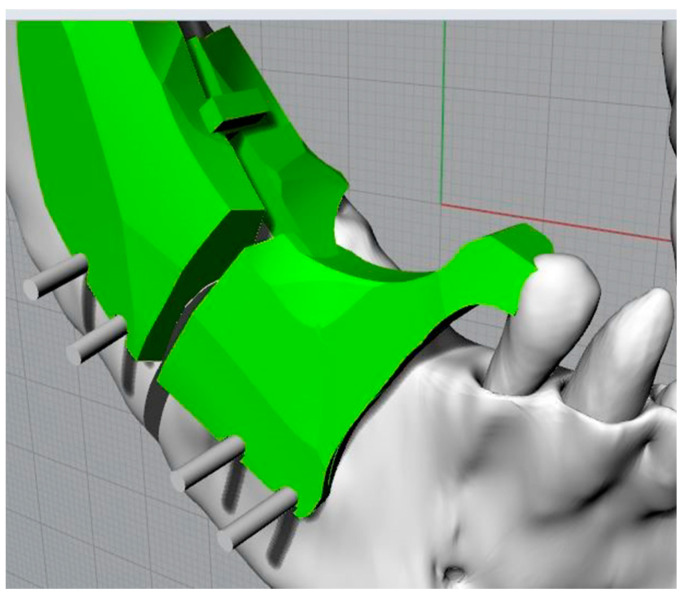
Cutting and repositioning guide (in green).

**Figure 12:**
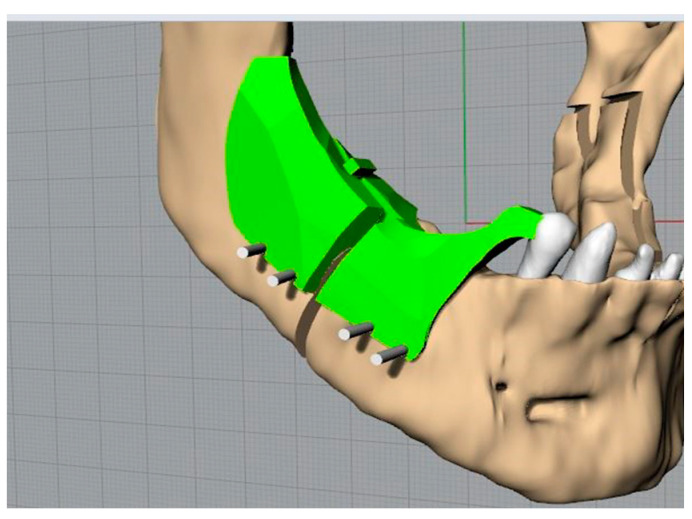
Placing the template and making the osteotomies.

Moreover, the thickness of the template could be reduced just choosing a different (more resistant) material to produce it by additive manufacturing. The template’s thickness in Figure 10, Figure 11 and Figure 12 has to be considered as an explanatory example.

The slots for the screws were defined through Boolean operations of difference between the template and the screws (Figure 11); grooves were also defined to allow easy removal of the template. 

The template, once physically created through rapid prototyping, was ready to be used in the surgical procedure. 

The visualization with beige colour was a screenshot of the simulation in the 3D environment of the surgery.

Once placed on the intact jaw, it was positioned (Figure 12) taking the last available tooth as a reference and identifying the points where the four screws had to be positioned, the head of which could be cut immediately using wire cutters or bone cutters. We then moved on to creating the osteotomies following the guide provided by the template. 

**Figure 13:**
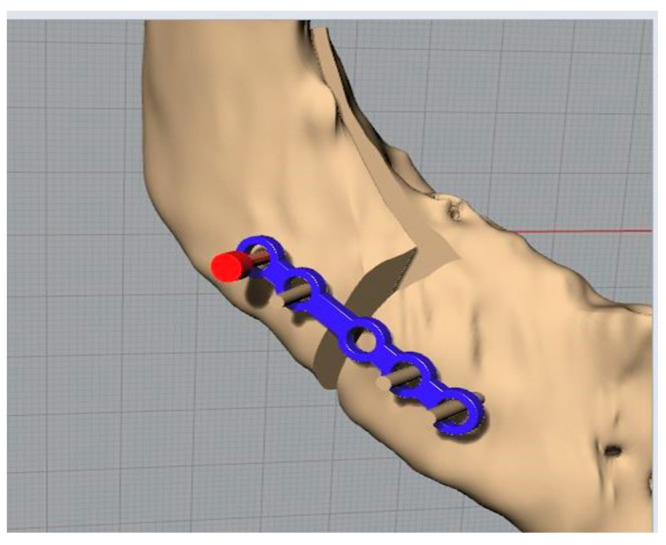
Replacement of the screws (in red the replaced one).

The template could now be removed. The repositioning of the jaw occurred thanks to the plate, which automatically led it to the final position; the only instruction provided to the surgeon, when using this five-hole plate, related to the pair of holes to be used for anterior fixation. At this point, the screws, one at a time, were removed using tools such as forceps, which were present in the operating room, and replaced with screws with heads that ensured definitive fixation (Figure 13).

The key steps in using the template during surgery were the cutting of the screw head and the automatic repositioning of the osteotomized elements using the template.

Cutting the screw head is an essential step since, in any case of surgery, blood flow is plentiful to allow for tracing of the holes.

**Figure 14:**
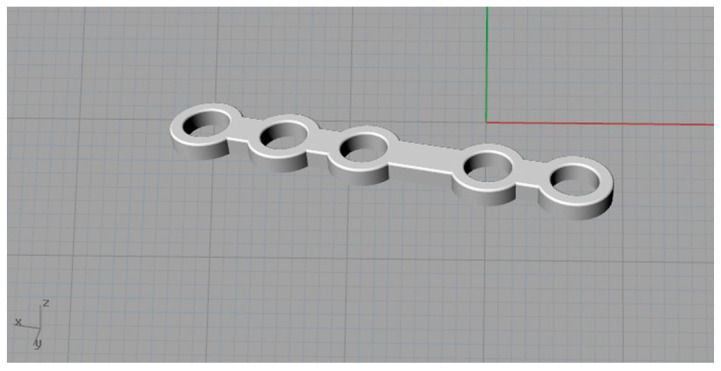
Perspective view of the plate.

The plate used for modelling was the ID—CAPFB3-2TM (Figure 14).

**Figure 15:**
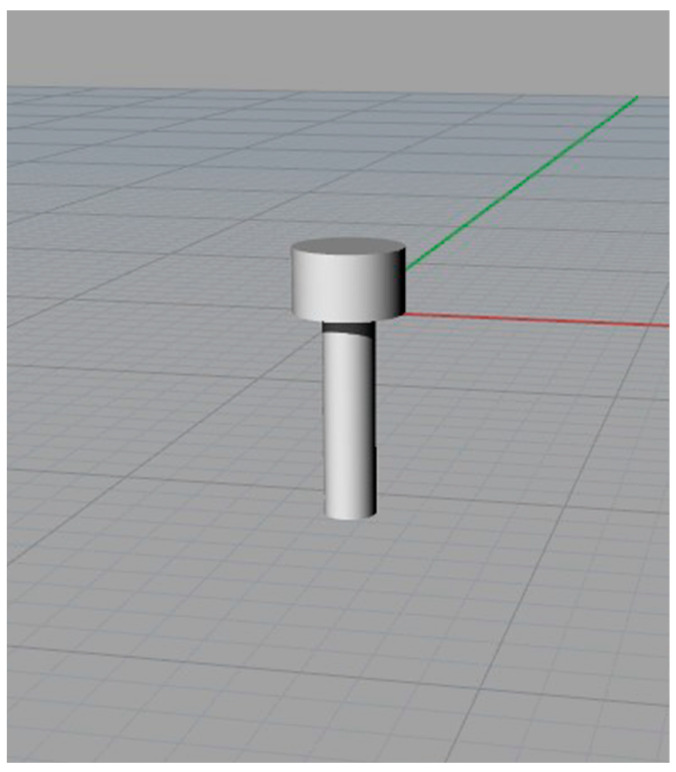
Perspective view of the screw.

After selection of the plate, the screws (Figure 15) that could be used had different body lengths (7, 8, 9, 11, 13, 15, 17, 19 and 21 mm).

The screw used during the generic BSSO intervention for which the templates were developed had a reduced body length; in this case, it was equal to 7 mm.

### 2.3. Manufacturing Models by Additive Methods

**Figure 16:**
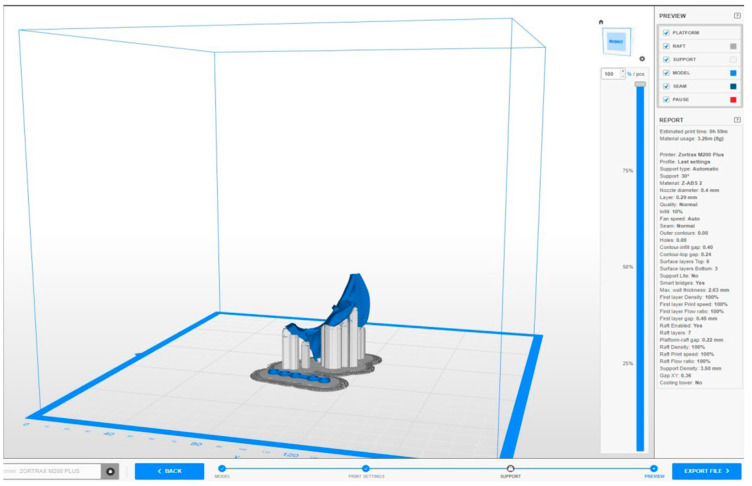
Z Suite’s work session.

**Figure 17:**
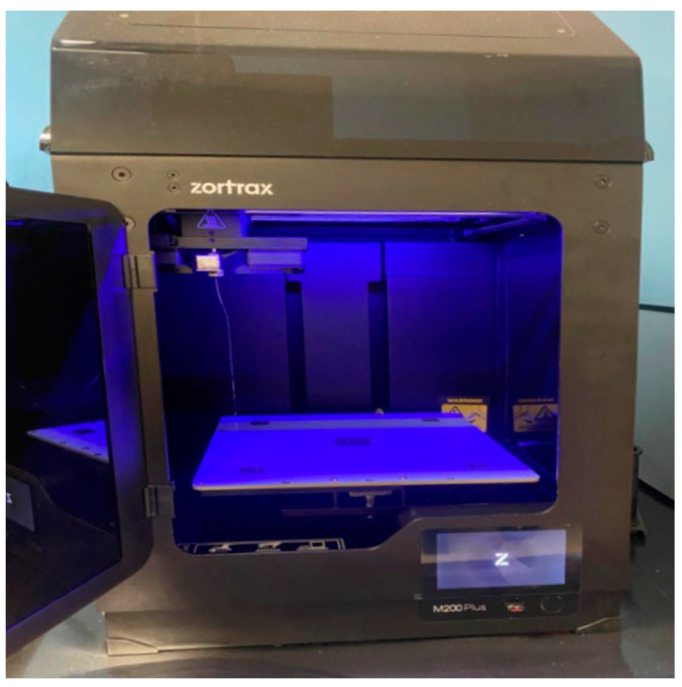
Zortrax M200 plus printer.

CAD modelling is therefore preparatory for printing; to print the digital model of the custom-made template, Z-Suite software was used, a slicing software package for Zortrax (Zortrax S.A., Lubelska 34, 10-409 Olsztyn, Poland) 3D printers (Figure 16). The printer model was the Zortrax M200 plus (Figure 17). 

These printers are compact, robust and professional, and usually offer good quality products, which makes them suitable for industrial design, dental and manufacturing industries. The material used was “Z-ABS 2”; this is a durable material that is suitable for prototyping and offers a semi-matt finish.

During the prototyping session, the plate was also printed, although the additive creation of the latter is not necessary, as the standard one can certainly be used too. It is good to remember that it is, in fact, an implantable element that must be subjected to greater sterilization and evaluations of the biocompatibility than the template, which is used only in the surgical setting to carry out the operation.

The height of the layers of material were as follows.

- Density of the first layer: 100%. This parameter refers to the percentage of material within the first layer (which, in this case, was set at 100%) and provides a completely full base which makes the structure robust.
- Density of the infill: 10%. This refers to the density of the infill of the part and affects the strength of the part and the printing time.
- Nozzle temperature: 250 °C. This refers to the temperature of the nozzle of the printing machine, which is usually between 230–260 °C.
- Build platform temperature: 100 °C. This parameter refers to the temperature of the print bed; a heated print bed is essential when printing with ABS, as it is an easily deformable material and is subject to shrinkage phenomena.

After the models had been sectioned on the software, the files were exported in the form of G-code and then transmitted to the printer. 

**Figure 18:**
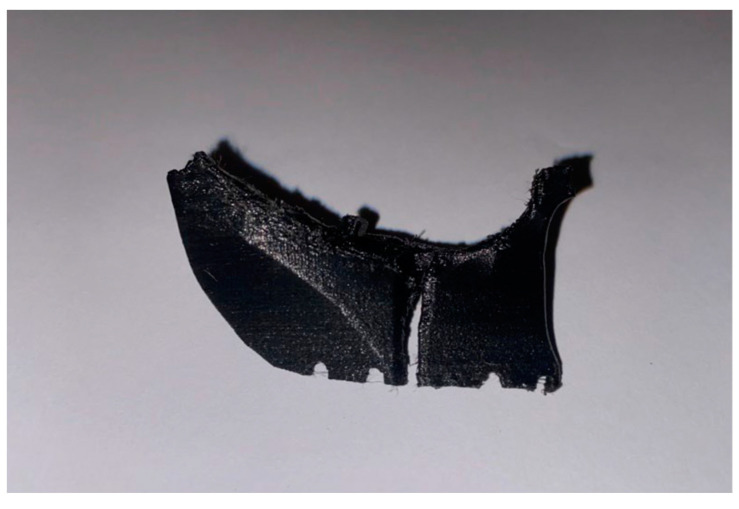
Custom-made printed template.

**Figure 19:**
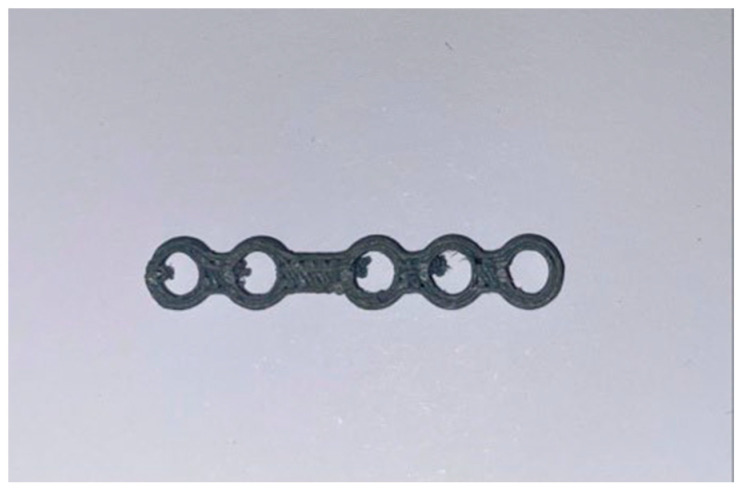
Printed plate.

The printed template and plate models are shown in Figure 18 and Figure 19. 

In this application, we decided to 3D-print the standard plate as well, although when carrying out normal surgical practices, the standard one from the kit can–and indeed must–be used; in fact, our decision was motivated by engineering-style exercises for evaluating and understanding the print quality.

For the purpose of carrying out the operation according to the methodology presented by [25], the plate present in the standard kits can also be used using the template presented here.

**Figure 20:**
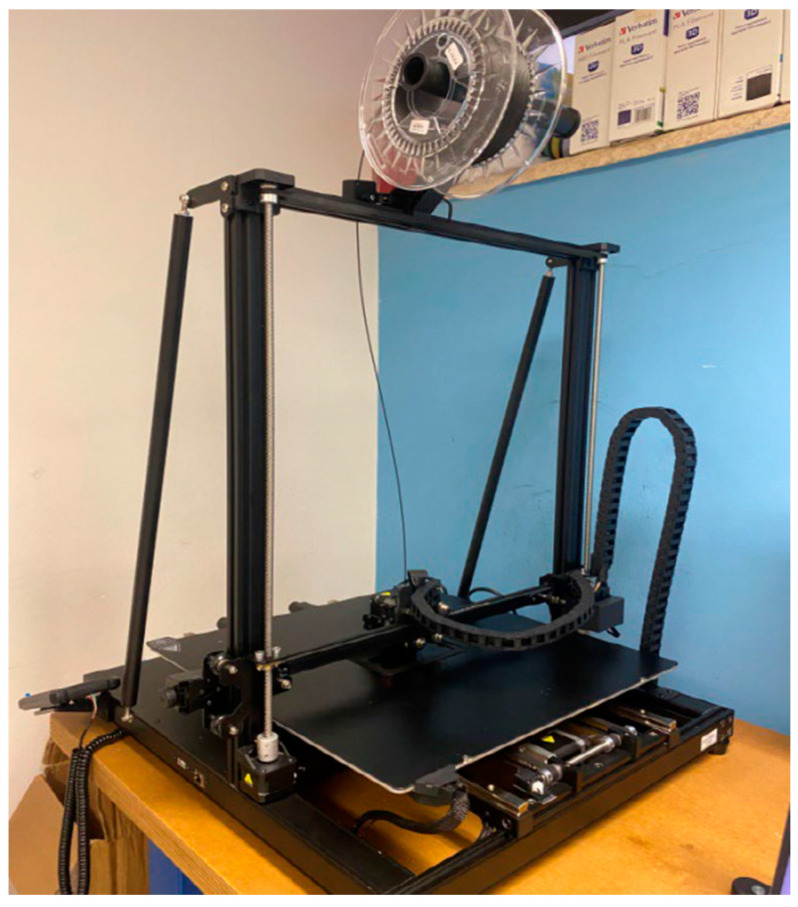
Creality CR-M4 printer.

For 3D-printing of the jaw, it was decided to print the osteotomized mandibular models to allow in vitro simulations. Therefore, the Creality Print CAM software was used to create the mandibular models. This is an FDM slicing software package produced and developed by the Creality company (18F, JinXiuHongDu Building, Meilong Blvd., Longhua Dist., Shenzhen, China 518131). In particular, in this case, a Creality CR-M4 printer (Figure 20) was used.

**Figure 21:**
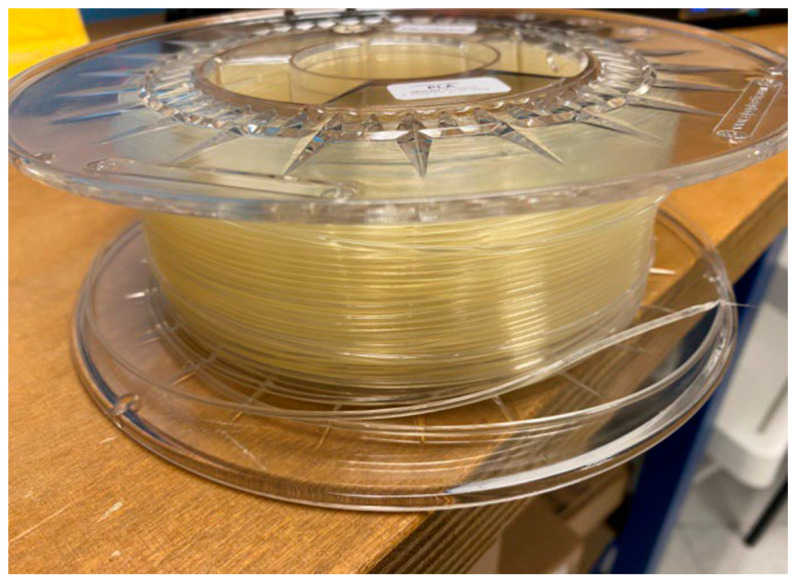
FIloalfa^®^ filaments.

The material used was FILOALFA^®^ PLA (polylactic acid) (Figure 21), a material also already used in biomedical contexts [26] manufactured by FILOALFA (Corso Duca degli Abruzzi, Torino, Italy). This is a biodegradable plastic obtained from renewable resources and therefore does not pollute the environment. It does not require a heated print bed, as it has good mechanical resistance and low retraction. Other features have been described in the previous paragraphs. As regards the surface finish, PLA is characterized by a notable surface shine and a perfectly smooth surface in which the layers are not visible, which makes it suitable for any type of application.

The authors of [27] collected information on how PLA has shown promise as a biomaterial in a plethora of healthcare applications, such as tissue engineering or regenerative medicine, cardiovascular implants, dental niches, drug carriers, orthopaedic interventions, cancer therapy, skin and tendon healing, and, lastly, medical tools/equipment.

The descriptive printing parameters of the print job performed here were as shown in Table 1, Table 2, Table 3, Table 4, Table 5 and Table 6; in particular in Table 1 the print quality parameters are shown, in Table 2 the kind of adhesion is specified, infilling parameters are in Table 3, speed parameters are in Table 4, Table 5 shows data about supports, and material processing parameters are in Table 6.

- Layer height: this is used to manage the thickness of each layer, for which the values, on average, are between 0.1 and 0.3 mm. The quality of additive printing is directly proportional to the processing time and inversely proportional to the height of the layer.
- Initial layer height: this is used to manage the thickness of the first layer.

**Table 2:** Adhesion of the build plate.

Adhesion of the build plate	Raft

This choice related to the type of adhesion to the print bed; that is, it matched several alternatives that permitted the piece to adhere to the print bed. In this instance, the raft—a real raft that serves as the piece’s base—was chosen with the intention of improving the model’s adhesion to the work area.

**Table 3:** Infilling parameters.

Density	20%
Line distance	2 mm
Pattern	Grid
Line multiplier	1
Percentage of overlap	30%
Overlap	0.16 mm
Layer thickness	0.20 mm

The infill consists of the material that is printed inside the object layer by layer. It allows the structure to be formed, guaranteeing support, and also determines its weight, surface finish and final resistance. Its main parameters are as follows.

- Infilling density: means the amount of material contained within the object (100%, completely full object; 0%, completely empty object). The filling density is directly proportional to the weight of the piece and the strength of the piece; for larger fillings, the printing times and the consumption of filament material increase. However, with a low filling percentage, the piece will be lighter and more fragile, even if it requires less time to be printed
- Infilling pattern: this refers to structural characteristics of the object and to the printing speed. The filling pattern adopted was the grid, which is the fastest, simplest and most solid filling variant.
- Percentage of overlap: this parameter controls the amount of overlap between the filling and the walls, expressed as a percentage. The higher the percentage, the greater the bond between the filling and the walls, so it is not recommended to set this parameter above 30%.

**Table 4:** Speed.

Infilling speed	50 mm/s
Outer wall speed	25 mm/s
Inner wall speed	25 mm/s
Top\bottom speed	30 mm/s
Support speed	60 mm/s
Travelling speed	100 mm/s
Print speed of the initial layer	15 mm/s
Travelling speed of the initial layer	25 mm/s

- Infilling speed: this means the filling speed; the default value was chosen.
- Travelling speed: this affects the quality of the piece and must be related to the printing temperature.
- Print speed of the initial layer: this refers to the speed of the first layer. Usually, this value is low to ensure greater adhesion of the piece to the printing surface.

**Table 5:** Supports.

Generated support	Yes
Support structure	Normal
Placement of the supports	Supports everywhere
Overhang angle of the supports	15
Pattern of the supports	Zig-zag
Density of the supports	30%
Z distance of the supports	

The supports have to be generated in order to support the parts that do not touch the printing surface.

- Support pattern: in this case, the default one was selected, namely, the zigzag one which is the easiest and simplest to remove.
- Support density: as for the filling, it was possible to choose the density of the supports.

**Table 6:** Material.

Printing temperature	200 °C
Initial printing temperature	200 °C
Initial printing temperature	200 °C
Build plate temperature	50 °C
Build plate temperature for the initial layer	50 °C

- Printing temperature: this varies primarily depending on the material used. In this case, FILOALFA^®^ PLA has an ideal extrusion temperature ranging across 190–210°. This temperature affects various aspects of the printed object: the dimensions (high temperatures for large objects), the adhesion of the piece to the printing bed (high temperatures and greater adhesion), the finish (low temperatures for a matte finish and high temperatures for a glossy finish), the layers’ size (less thickness, higher temperatures).
- Initial printing temperature: it is often useful to increase the temperature of the first printing layer to increase the adhesion of the first layer to the printing surface.
- Build plate temperature: this parameter refers to the temperature of the printing bed and is an equally important parameter, since the correct temperature allows one to avoid warping and adhesion problems in the piece. Some materials require particular temperatures to ensure greater adhesion; in this case of PLA, it is advisable to have a printing plate with a temperature between 40–50° but also lower temperatures due to the fact that this material tolerates the cooling phase well and therefore does not usually present warping problems.

**Figure 22:**
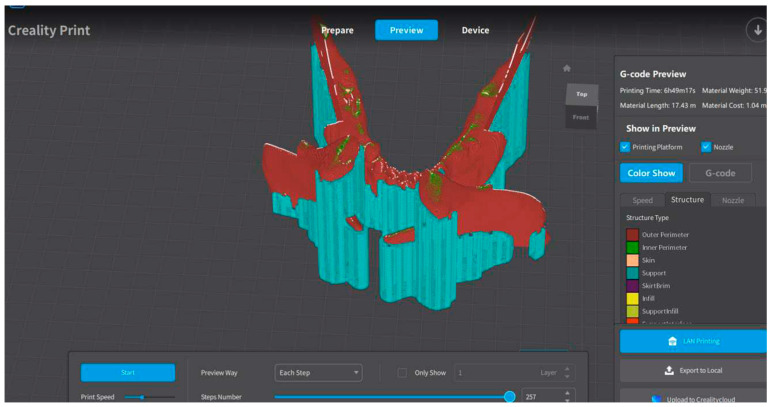
Creality’s work session.

Creality (Figure 22) was used to create the object when it was about to be printed.

**Figure 23:**
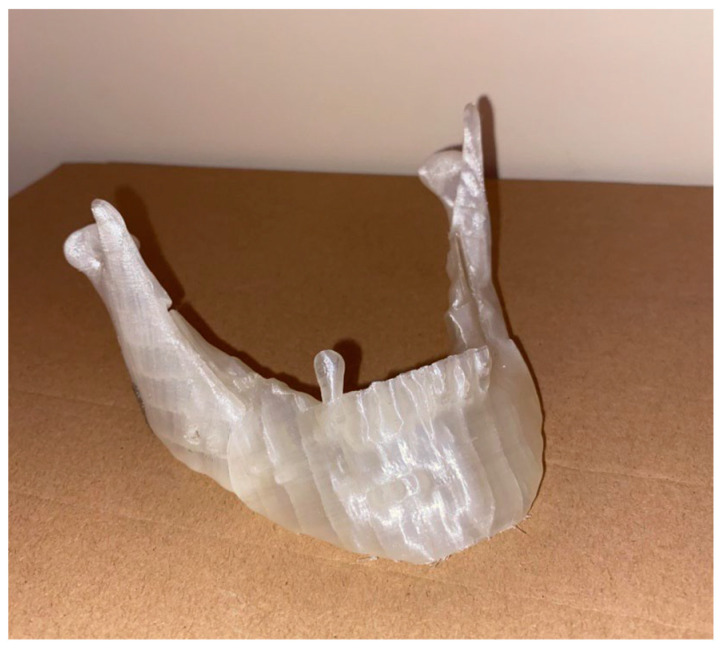
A 3D model of the jaw.

Once the models had sectioned, the file was exported in the form of G-code and was transmitted to the printer. The printout of the mandibular models is shown; a 3D print of the mandible was made in order to show the positioning and the template of the mandible assembly, as shown shortly in Figure 23.

## 3. Results

In the realm of medicine and surgery, simulation is crucial, since it is a technique that makes it possible to accomplish goals with increased accuracy and precision. The purpose of the simulation is to recreate a scenario and test the validity and feasibility of what has been designed and budgeted for potential real use in the medical field. Its application in the medical industry and other related fields guarantees increased safety and accuracy during the surgical process, preventing mistakes and lessening the severity of any consequences. 

For these purposes, we proceeded with the in vitro simulation of the orthognathic surgical procedure using a custom-made template designed and printed in 3D. It is necessary to specify that in the case in question, the patient was suffering from a prognathic syndrome for which, in the planning phase of the mandibular distraction, a 3 mm advancement of the mandibular segment was set. Furthermore, the available template was designed in such a way as to rest on the last tooth available during the operation. For rigid internal fixation, however, a standard titanium plate with five holes was used.

**Figure 24:**
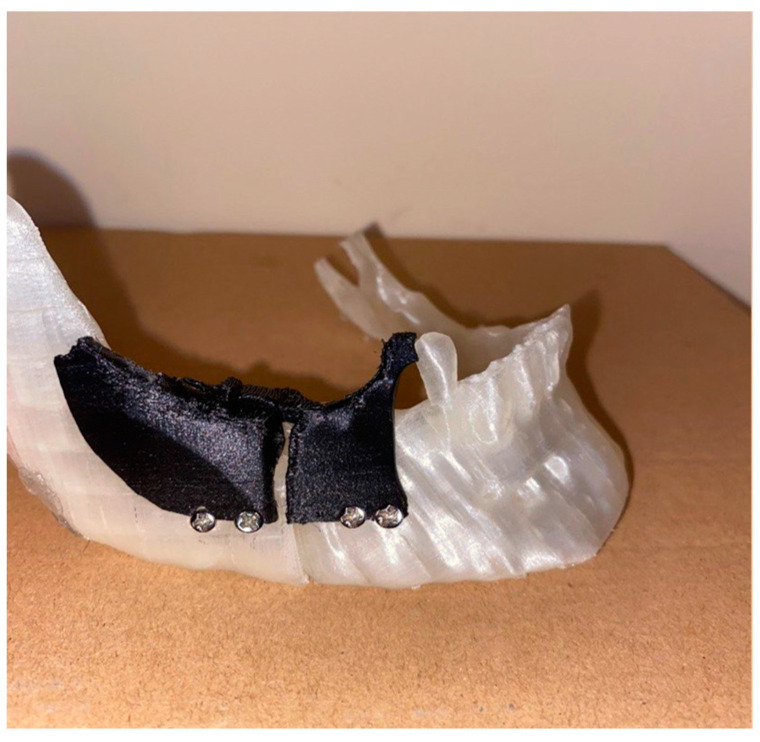
Positioning of the template and screws.

During the actual surgical procedure, as the template that was physically created through rapid prototyping was available, this was placed on the intact jaw of the patient, having the last tooth as a reference (Figure 24), which allowed us to identify the points where the screws must be positioned, then their heads could be cut using wire cutters.

**Figure 25:**
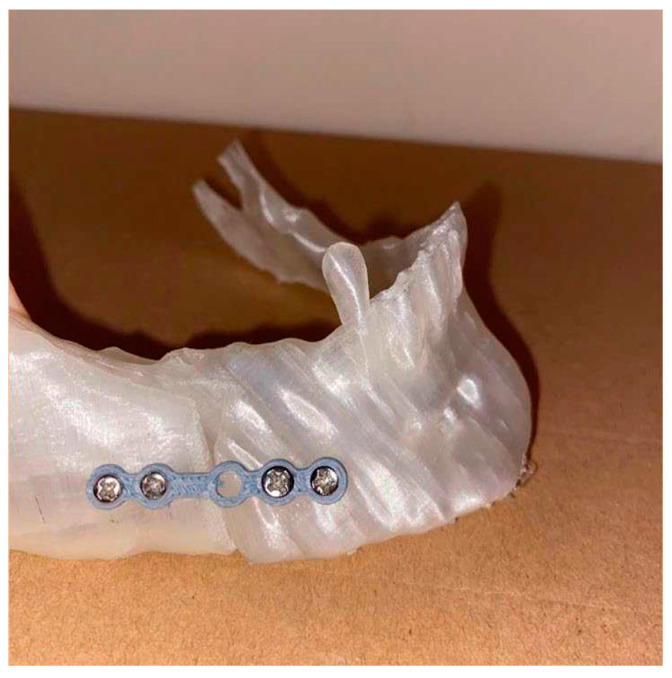
Final position of the jaw with the plate and screws.

**Figure 26:**
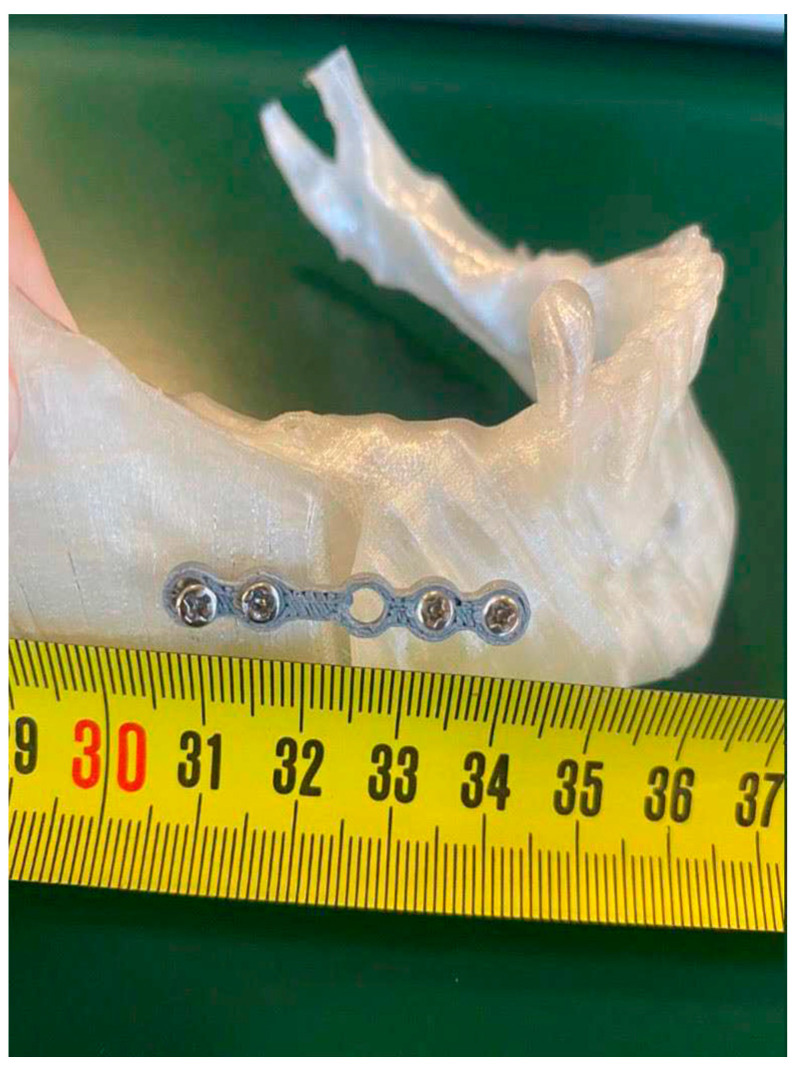
Dimensional verification of mandibular advancement.

After inserting the screws, we continued with the creation of the osteotomies, following the guide provided by the template. Later, the template was removed, and the repositioning of the mandible occurred as planned thanks to the use of the plate, which automatically led it to the final position. At this point, the screws were removed, again using tools such as pliers, and replaced by screws with heads that guaranteed definitive fixation (Figure 25). The dimensional verification of the 3 mm mandibular advancement is represented below (Figure 26).

**Figure 27:**
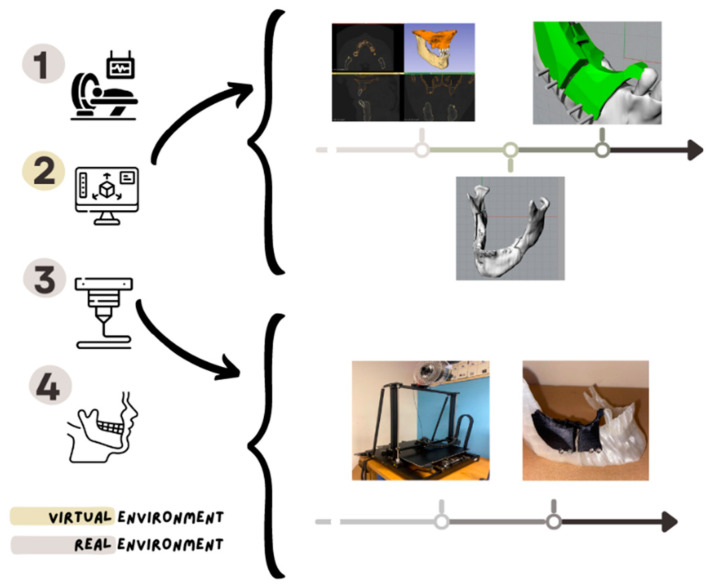
Flowchart of the new procedure for designing and manufacturing the proposed low-cost custom-made template for BSSO with standard plates.

In order to summarize the 14-step procedure in four macro-steps, a flowchart of the activities and operations is reported in Figure 27, in which Macro-step 1 comprises Steps 1 to 5, Macro-step 2 comprises Steps 6 to 10, Macro-step 3 comprises Steps 11 and 12, and Macro-step 4 comprises Steps 13 and 14.

## 4. Discussion and Limitations

The steps presented so far have demonstrated the possibility of reproducing and designing a template in a virtual environment, following a pre-defined methodology [25] according to a low-cost and custom-made approach. In fact, the possibility of having a template that perfectly fits the mandibular morphology, and more generally to the anatomical elements of the maxillofacial region, allowed us to improve the quality of the intervention. Moreover, as reported by [28], once the staff have been properly trained, it is possible to achieve even shorter timeframes for planning surgical cases. 

Many studies have compared computer-assisted planning with classical planning and found favourable results in terms of accuracy in all bony segments for computer-aided planning [29]. 

The virtual environment allows one to plan the type of intervention and, therefore, the osteotomies to be carried out directly in the virtual environment without having to go to the articulator; the template created in FDM represents the final solution created by the surgeon together with the engineer or technician who deals with the three-dimensional modelling part. Patient-specific cutting guides, in fact, allow accurate repositioning of the mandibular segments [30]. With the template presented in this article, in fact, the aim was to pre-define an operative procedure for the study, and its realisation and use in a real case. The study and analysis of the surgical case in a three-dimensional environment led to both planning and realisation by decreasing the probability of error that would have occurred with the traditional approach. Nevertheless, the costs, effort and times to design, test and 3D print the custom-made templates and plates are somewhat high; thus, the proposed solution fits with all the surgeons’ needs during pre-planning, testing and executing the BSSO surgery, using a virtual environment for planning, low-cost 3D-printed templates for testing and standard plates for surgery.

Considering the trend in the field of orthognathic surgery, one cannot fail to consider that machine learning can be used increasingly, as reported by [31], for the prediction of postoperative skeletal changes to reduce the surgeons’ workload. The purpose of the work of [32] was to explore a deep learning-based, reliable and automatic approach for predicting the repositioning vectors of the mandible in an orthognathic surgical plan. Once one has predicted the repositioning vectors, the proposed methodology allows one to create a physical mock-up of the patient’s mandible and of the templates used to test the surgery. The novelty of the proposed method can be found in the use of standard plates for the physical simulation and in the low costs of the materials and methods used for the physical representation of the intervention. Even if the size of the guide seems to be too big to be used, periosteal elevation is not so larger than in conventional BSSO procedure and is irrelevant from a surgical point of view and identical to other commonly adopted custom-made guides’ procedures. Furthermore, there is no need to guide these osteotomy lines in the procedure presented here because they refer to screw holes’ positioning after VSP in relation to the dimensions of the standard plates achieved in the procedure. Moreover, positioning of the buccal osteotomy line is included in the guide even if it is not relevant for the precise positioning of the fragment, because it is related to the holes’ position and not to fitting the surface margin’s fragments. The buccal osteotomy line is not relevant for positioning the fragments because it is horizontally placed in the same direction of the fragments’ movements after BSSO and, for this reason, it is not guided in any positioning technique (traditional occlusal splints, custom-made guides or custom-made guides and plate-based techniques). Finally, the amount of cortical bone removed after mandibular setback is precisely driven by the overlapping of the ramous cortical bone on the fragment of the mandibular arch, depending on the amount of setback, and the nerves’ safety is not related to the guide for positioning the osteotomy’s line but to the osteotomy’s depth in line with the correct surgical technique (not depending on the use of template).

From a technical point of view, the most important errors and limitations were found in the imperfect arrangement of the condyles and in the poor predictability of the soft tissue’s movements and their influence on the joints.

It must also be considered that unlike the traditional method, where the procedure is completely managed by the surgeon, in order to follow this approach, a close collaboration must be established between the technician and physician, who must work together proactively.

Finally, in a patient with a more developed dental arch than the described test case, the surgical practice would be similar. In fact, the template developed through a CAD/CAM approach can continue to be customised and adapted to the patient’s morphology; the only substantial difference lies in the different extension of the mandibular body and the shape of the interface with the last available tooth. In anatomical conditions in which no teeth are available (non-dentulous patients), it would be necessary to evaluate, together with the surgeon, the way to proceed and the reference to take (for example, using an implanted pin in the mandible), but this case was not investigated in this study. Nevertheless, the heart of the methodology continues to lie in the use of screws (whose heads have been cut off) for automatic relocation of the mandible using the standard plate.

## 5. Conclusions

So, where is orthognathic surgery headed? Considering the trend in the field of orthognathic surgery, the custom-made approach can only be considered the winning choice, not only from a surgical point of view, with better planning and more precise pre-operative evaluation, thanks to innovative tools and approaches that have cutting edge performance, as expressed by [33,34,35,36,37], but allows the patient to be increasingly satisfied not only from a functional point of view, with a clear improvement in the features that concern the maxillofacial area, but also from an aesthetic point of view; in fact, we can have a greater awareness of what will be the morphology of the bimaxillary compartment following the intervention.

A non-trivial aspect to consider concerns the time–cost–quality triad. The combination of the latter two represents one of the major strengths of this new approach. In the proposed methodology, the designed template allows us to combine all the benefits of a custom-made device while optimizing the costs of a personalized approach thanks to the use of one template for both cutting and repositioning and, above all, thanks to the special procedure that allows us to use standard plates instead of very costly and time-consuming (during their design and manufacture) custom-made plates.

As described in scientific papers over the last 10 years, and in the proposed methodology, the personalized approach starts from the manipulation of the structures of the mandibular bone in a 3D environment. Furthermore, the manufacture of implantable biocompatible custom-made metal devices which require longer design and manufacturing times is not required; for custom-made plates that adapt perfectly to the bone’s morphology, an evaluation of the loads and mechanical resistance may be necessary; thanks to the use of standard plates, this issue is completely deleted and no additional studies are needed to use them in a BSSO surgery. Furthermore, the difference in the cost of custom-made titanium plates for internal fixation is approximately 10 times greater than that of standard ones, as reported by [38], so the proposed approach is very cheap compared with the standard one.

The approach presented in this article has great advantages not only from the point of view of pure planning; in fact, as has been widely said, virtual simulation allows an earlier and timely evaluation of the surgical case to be treated, but this case was managed as a training model [39].

Nowadays, the potential of a computer-aided surgical approach lies not only in a better planning phase but also in optimization of the quality standards and also in better performance in terms of lower doses of radiation and financial costs [40].

As regards the training of personnel, we must start from the following assumption. The definition of the extent of the osteotomy is evaluated in synergy between the surgeon and healthcare personnel and is then communicated to the engineer or, more generally, to the technician who has to deal with CAD modelling and subsequent additive manufacturing. 

The proposed methodology also allows us to create low-cost physical mock-ups to simulate the BSSO surgery in order to train surgeons for real surgeries. Again, the use of standard plates allows to minimize the costs of training surgeons for very non-standard cases with models of bones from real patients.

An approximated estimation made by three maxillofacial surgeons allowed us to assess the reduction in the pre-operative planning time (pre-orthodontic analogical analysis, pre-surgical cephalometric analyses, surgical VTO (cephalometric programming), taking pre-surgical impressions, assembly in the articulator, segmentation of the models, preparation of the templates and post-surgical verification), which was from 3 person-days to about 1.5 person-days, and a reduction in the costs in Campania (Italy) from about EUR 4000 (for a customized template and customized plates) to about EUR 1000 (for a customized template with standard plates).

Finally, given the general trends in the IT field, with a special focus on the surgical field, one could think of using AI and machine learning techniques to make some of the programming and evaluation phases of the intervention semi-automatic.

In orthognathic surgery, the application channels of artificial intelligence have been identified by [41] for improving the diagnostic precision with AI-enhanced maxillofacial imagery, planning treatment using 3D models and CAD/CAM manufacture of custom orthodontic and surgical appliances.

Moreover, VSP appears to be a more accurate method for planning orthognathic treatment and the accuracy of splints in favour of CAD/CAM splints; even the duration of an operation using VSP is significantly reduced [42].

As expressed by [43], in fact, AI aims, in this sector, to support professionals in their instrumental assessments in order to achieve better quality standards; in fact, its applications in OGS are mostly based on a deep learning architecture and machine learning.

## Figures and Tables

**Table 1:** Print quality.

Layer height	0.20 mm
Initial layer height	0.20 mm

## Data Availability

Full data set about the template’s Clinical trial is available publicly on demand at Italian Health Ministry, but not sharable publicly. Data related to the simulated tests on real patients are available on request because, even if the informed consent has been signed and data have been anonymized, the Italian law doesn’t allow to share publicly the data set.
